# What Is Acute Myeloid Leukemia?

**DOI:** 10.1002/jha2.70063

**Published:** 2025-05-25

**Authors:** Daniel Mazza Matos, Eduardo Magalhaes Rego

**Affiliations:** ^1^ Flow Cytometry Division Cell Processing Center (CPC) Center of Hematology and Hemotherapy of Ceará (HEMOCE) Fortaleza Brazil; ^2^ Faculty of Medicine University of Fortaleza (UNIFOR) Fortaleza Brazil; ^3^ Faculty of Medicine of the University of São Paulo Department of Clinical Medicine of the Faculty of Medicine of USP University of São Paulo São Paulo Brazil

## Abstract

**Background:**

The recently published fifth edition of the World Health Organization (WHO) Classification and the International Consensus Classification (ICC) of myeloid neoplasms diverge in their definitions of acute myeloid leukemia (AML). Fundamentally, this current situation is problematic for two main reasons. First, the disagreement between the WHO and ICC reflects a conceptual conflict regarding the necessary and sufficient conditions for diagnosing AML. This leads to some confusion in areas such as nomenclature, therapeutic eligibility, clinical trial selection, and guidelines development. Second, in clinical practice, the two systems may assign different diagnoses to the same patient with a single disease entity. Such inconsistencies are unacceptable and highlight the urgent need for harmonization between the two classification systems.

**Topic:**

In response, we put forward a proposal based on an unorthodox approach that combines philosophical reflections on essential and accidental properties with the latest clinical evidence concerning “AML subtypes with defining genetic abnormalities,” with the aim of initiating the unification of the WHO and ICC classification systems.

**Implications:**

In this review, we propose a preliminary recommendation for a unified WHO‐ICC classification of five AML subtypes: AML with *PML*::*RARA* rearrangement, AML with *NPM1* mutation, AML with *KMT2A* rearrangement, AML with *MECOM* rearrangement, and AML with in‐frame bZIP *CEBPA*. We hope that these initial efforts can be followed by a more definitive attempt to unify the WHO and ICC.

**Trial Registration:**

The authors have confirmed clinical trial registration is not needed for this submission.

## 1 Introduction

What is acute myeloid leukemia (AML)? This is a strange question, we admit. The question, it is important to note at the outset, is not what it means for a person to be diagnosed with AML. The real question is, “What is acute myeloid leukemia?”

Readers who are now faced with a question of this sort may be saying to themselves: “Well, what a silly question! AML is what is defined as AML.” So, simply defining the technical term “acute myeloid leukemia” is, at first glance, all that is required to classify and understand what AML is. This is correct, let it be said at once, although it must also be acknowledged that there is a certain naïveté in assuming that definitions can be obtained without difficulty. We will discuss this point in greater detail in the sections that follow.

At the outset, it is important to clarify that this article does not aim to examine the historical development of hematopoietic neoplasm classification [1, 2]. Instead, our focus is on the category “AML with defining genetic abnormalities,” [3] also called “AML types with recurrent genetic abnormalities” [4]. For the sake of consistency, from now on we will refer to this group of diseases by a neutral name (neither WHO nor ICC): “AML subcategory with defining genetic abnormalities.”

## 2 The 5th Edition of the World Health Organization Classification (WHO) and the International Consensus Classification (ICC) for AML

AML subcategory with defining genetic abnormalities was first introduced in the WHO classification (commonly referred to as the “blue book”) in its inaugural edition, published in 2001. In June 2022, a preliminary article outlining the fifth edition of the WHO classification was released [3] (although the finalized version in book format was not published until 2024). Two months later, in September 2022, a separate group of experts specializing in the clinical, pathologic, and genetic aspects of myeloid neoplasms published (for reasons that will not be discussed here) their own independent systematization: the International Consensus Classification (ICC) [4]. Notably, the WHO and ICC offer differing perspectives in response to a fundamental question: “What is AML?”

According to the updated book version of the WHO classification (WHO Classification of Tumours Editorial Board. 2024. Haematolymphoid Tumours. 5th ed. International Agency for Research on Cancer), the diagnosis of the AML subcategory with defining genetic abnormalities does not require a minimum blast percentage, albeit it is said to be essential an “increase of peripheral blood and/or bone marrow blasts.” The ICC, in turn, have a different opinion: in its classification, two blast thresholds are proposed for defining this AML subcategory, namely, a minimum of 10% blasts and, in certain contexts, at least 20% blasts [4]. The ICC classification differs from the WHO not only with respect to blast thresholds, but also in terminology and in the number of entities included in this group of diseases [5, 6]. It is true that the WHO and ICC agree that the genetic characterization of AML is the best predictor of therapeutic response and clinical outcome than the imprecise morphological quantification of myeloblasts [7]. Nonetheless, the existence of two divergent diagnostic criteria for the AML subcategory with defining genetic abnormalities, each endorsed by a different group, highlights the current lack of a standardized definition for this group of diseases.

Is this really a problem? This is not a simple question to answer. On one hand, in the routine practice of hematologists, genuine cases of diagnostic disagreement (due to their rarity) are likely to be subjects of theoretical debate rather than practical concern, rendering them more a matter of academic interest than a challenge in day‐to‐day clinical work. For example, cases with less than 10% blasts and *PML::RARA*, *RUNX1::RUNXT1*, and *CBFB::MYH11* rearrangements are exceedingly uncommon [8]. In addition, a recent study showed that when the WHO and ICC classifications were compared, only 1.3% of AML cases exhibited “major diagnostic discrepancies”—defined as discrepancies leading to different diagnoses with significant and clear therapeutic implications [9].

On the other hand, while at first glance it may seem that eventual diagnostic divergences are of little clinical impact from a practical point of view, it is important to remember that it involves the issue of whether to give a patient the dramatic diagnosis of AML. A cancer diagnosis has long been associated with depression and anxiety [10] and, for a layperson, receiving the stigmatized diagnosis of AML is emotionally distinct from receiving a diagnosis of myelodysplastic syndrome (MDS), a disease that remains largely unknown to the general public.

There are two alternatives to solve this problem. The first is to accept both classifications and, in the event of clashing diagnoses, treat patients according to the best available therapy. This seems to be the option advocated by Platzbecker et al. [11], although it is crucial to emphasize that some of the cases the authors mention in their article merely differ in the nomenclature used by the WHO and ICC. The second alternative is to seek a unified definition for entities that are defined differently across the two classification systems. We believe this is the preferable alternative for two main reasons. First, the coexistence of two competing classification systems creates confusion regarding diagnostic terminology, therapeutic eligibility, clinical trial inclusion, and the development of clinical guidelines. For example, the 2024 Brazilian consensus on acute promyelocytic leukemia (APL), which bases its recommendations on international guidelines, does not clearly define the minimum blast percentage required for an APL diagnosis [12]. Second, the conundrum generated by the inconsistencies between the WHO and ICC should not be tolerated because, once accepted, we end up facing paradoxical situations where the same disease entity would and would not be itself at the same time. Consider, for a moment, a patient with evidence of anemia, megakaryocytic dysplasia, 7% bone marrow blasts, and alterations in *DNMT3A*, *NRAS*, and *NPM1*. The WHO does not specify a lower blast limit for AML subcategory with defining genetic abnormalities, although the text of the “blue book” states that an “increase in blasts in the peripheral blood and/or bone marrow” is essential for the diagnosis. Traditionally, more than 5% in bone marrow or 2% in the blood have been considered an abnormal percentage of blasts. Thus, according to the WHO, the diagnosis for this case is AML with mutated *NPM1*. However, according to the ICC, since the blasts percentage is less than 10%, this patient has a diagnosis of MDS, not otherwise specified (NOS) [8, 9]. This is a very strange situation—not to say that this ambiguity of diagnoses indicates that there is something profoundly wrong with the acceptance of two concurrent classification systems—because the patient in question clearly has a single disease, which cannot simultaneously be classified as both AML with mutated *NPM1* and MDS, not otherwise specified (NOS).

So, what we have here is a problem of *definition*. And since the divergence between the WHO and ICC concerns on how to define entities of the AML subcategory with defining genetic abnormalities, in the following Sections 3 and 4 we will discuss briefly what it means to define an entity. These sections are important to show the perspective from which nosological entities are defined. In effect, the medical classification of hematological malignancies—more especially, AML subcategory with defining genetic abnormalities—is covered, a subject that has received little attention in the literature. In Section 5, which is the core of the article, lies our suggestion for the unification of both classification systems, at least for some entities for which we believe there is sufficient evidence. Section 6 is the conclusion of the article.

## 3 Defining Entities of the World

What is a tiger? A tiger is an animal that possesses characteristics allowing us to distinguish it from, for example, lions or panthers. However, this type of identification offers only a general description. What concerns us here are the *criteria* by which something is defined as a tiger. Let us put the problem in another perspective. Consider, for a moment, an ordinary tiger. Now, suppose its stripes are removed—does it remain a tiger? Next, its tail is removed. Is it still a tiger? Then imagine the removal of its whiskers, teeth, and claws. Is it still a tiger? At this stage, many might hesitate to continue affirming that it is [13]. Defining things is a curious task because, in everyday life, it is assumed that everyone can identify and define a tiger, even if they do not know the precise definition of the term. Problems arise, however, when precise definitions are required. For example, what is the definition of “bird”? One might define a bird as an animal that flies. This definition of bird encompasses many entities that clearly fall under the category of birds, such as canaries, owls, and robins. But it is problematic because a penguin is a bird, even though it does not fly. And, to muddy the waters, a butterfly is not a bird even though it is an animal that flies.

The problem with concepts such as tiger, bird, game, rheumatoid arthritis, AML, and so on, is that we habitually search for a single set of features that are necessary and sufficient to categorize them, but these features are not easy to find. This set of privative features constitutes the essence of the subject to which the terms refer. This concept is the focus of the philosophical view known as “essentialism,” which holds that certain properties are essential to the identity of objects [13, 14]. What we know about “essentialism” is that, apart from mathematical and geometrical objects, defining things is inherently challenging [14]. And because diseases are conceptual constructs that we define for the purpose of providing a sense of order and understanding in our daily clinical practice [15], their definitions are also problematic. For example, when we ask, “what is AML?” we expect an answer that defines AML and, ontologically, we seek to uncover the essence of what AML truly is through its definition. But what does “definition” actually mean?

“Definition of a disease” refers to a nominal definition of a disease category—that is constituted by a set of criteria (necessary and sufficient conditions) which a concrete case of disease must fulfill in order to fall into that particular category [16].

With a definition in hand, it becomes possible to construct a classification system for diseases upon which clinical diagnoses are based [16]. In the case of hematopoietic neoplasms, a precise definition of each entity is crucial because, in its absence, both nosology and treatment of these serious diseases are profoundly compromised [2]. Therefore, a precise definition for AML is essential. The key to obtaining this definition lies in selecting “the necessary and sufficient conditions,” [16] specifically the conditions required for diagnosing the AML subcategory with defining genetic abnormalities. In the following section, we will examine whether these conditions can be identified.

## 4 AML Subcategory With Defining Genetic Abnormalities: How to Define This Group?

Let us start by understanding what an essential characteristic or property of an entity is. In general terms:

*P* is an essential property of an object *o* just in case it is necessary that *o* has *P*, whereas *P* is an accidental property of an object *o* just in case *o* has *P*, but it is possible that *o* lacks *P* [13].

The assumption considers the relationship between a property *P* (that can be essential or accidental) and an object *o*. Accidental properties are those that some object may or may not have, while essential properties are necessary properties. Essential properties are required and cannot be lost: if you “remove” an essential property you are no longer describing the same object. Let us consider an example: for some years now, we have been wondering whether the presence of CD5 antigen (the property) on abnormal B‐cells would be an essential or an accidental property for the diagnosis of chronic lymphocytic leukemia (CLL) (the object) [13]. Thus, we realize that Aristotle's notions of essential and accidental properties^i^ might be useful in systematizing the relationship between CD5 expression and CLL, as we have discussed in detail elsewhere [13]. Our conclusion, based on currently available data, is that CD5 antigen positivity is a sine qua non condition for the diagnosis of CLL—in other words, CD5 expression is an essential property of CLL [13, 17]. The same line of reasoning can be applied to AML subcategory with defining genetic abnormalities, in these terms:

The genetic abnormality (*P*) is an essential property of AML subcategory with defining genetic abnormalities (*o*) just in case it is necessary that AML subcategory with defining genetic abnormalities (*o*) has the genetic abnormality (*P*), whereas the genetic abnormality (*P*) is an accidental property of AML subcategory with defining genetic abnormalities (*o*) just in case AML subcategory with defining genetic abnormalities (*o*) has the genetic abnormality (*P*), but it is possible that AML subcategory with defining genetic abnormalities (*o*) lacks the genetic abnormality (*P*).

In analyzing the proposition, it becomes clear that, since it is not possible for the AML subcategory with defining genetic abnormalities to exist without the corresponding genetic abnormality, this abnormality is an essential property for diagnosing this category. However, this proposition does not aid in defining the group, as it is tautological. The tautology arises for an obvious reason: the stipulated definition of the entity AML subcategory with defining genetic abnormalities already presupposes the presence of the property “genetic abnormality.”

In any case, up to this point, the WHO and ICC are in agreement. Moreover, they concur on another key point: the property “genetic abnormality” is necessary for diagnosis, though not sufficient on its own. This may not appear prima facie obvious, but the rationale behind this implicit assumption by both the WHO and ICC is that genetic abnormalities typically associated with AML have also been identified in individuals without any clinical evidence of the disease. For example, the *BCR::ABL* fusion gene, the mixed lineage leukemia gene (*MLL*), and even *PML::RARA* and *CBFB::MYH11* rearrangements were showed to be present in healthy individuals [18, 19]. The lesson to be remembered from these findings is the now long‐standing knowledge that genetic abnormalities, the so‐called “driver mutations,” [1] cannot really by themselves “drive” the process of leukemogenesis to term, and malignant transformation also requires the emergence of secondary genetic events and/or loss of immune surveillance.

So, what is the point of disagreement between the WHO and the ICC? “Blasts,” this is the answer [20, 21, 22]. The WHO does not consider necessary to formally require a minimum percentage of blasts to establish the diagnosis of AML subcategory with defining genetic abnormalities, except in two subtypes: AML with *BCR*::*ABL1* and AML with *CEBPA* (although it is said to be essential the increase in “peripheral blood and/or bone marrow blasts”) (Table 1). The ICC, in contrast, considers the presence of a minimum blast percentage a secondary but necessary criterion, with certain exceptions (Table 2). Specifically, the WHO and ICC differ on whether the ideal threshold for blasts should be 20%, 10%, or simply an “increased percentage”—their disagreement centers on defining a *blast threshold*.

**TABLE 1:** WHO definition of AML with defining genetic abnormalities.

Acute myeloid leukemia with defining genetic abnormalities
Acute promyelocytic leukemia with *PML::RARA* fusion^a^
Acute myeloid leukemia with *RUNX1::RUNX1T1* fusion^a^
Acute myeloid leukemia with *CBFB::MYH11* fusion^a^
Acute myeloid leukemia with *DEK::NUP214* fusion^a^
Acute myeloid leukemia with *RBM15::MRTFA* fusion^a^
Acute myeloid leukemia with *BCR::ABL1* fusion^b^
Acute myeloid leukemia with *KMT2A* rearrangement^a^
Acute myeloid leukemia with *MECOM* rearrangement^a^
Acute myeloid leukemia with *NUP98* rearrangement^a^
Acute myeloid leukemia with *NPM1* mutation^a^
Acute myeloid leukemia with *CEBPA* mutation^b^
Acute myeloid leukemia, myelodysplasia‐related^b^
Acute myeloid leukemia with other defined genetic alterations^a^

^a^ Increase bone marrow and/or peripheral blood blasts required.

^b^ At least 20% of blasts.

**TABLE 2:** ICC definition of AML types with recurrent genetic abnormalities.

Classification of AML
Acute promyelocytic leukemia (APL) with t(15;17)(q24.1;q21.2)/*PML::RARA* ^a^
APL with other *RARA* rearrangements^a^
AML with t(8;21)(q22;q22.1)/*RUNX1::RUNX1T1* ^a^
AML with inv(16)(p13.1q22) or t(16;16)(p13.1;q22)/*CBFB::MYH11* ^a^
AML with t(9;11)(p21.3;q23.3)/*MLLT3::KMT2A* ^a^
AML with other *KMT2A* rearrangements^a^
AML with t(6;9)(p22.3;q34.1)/*DEK::NUP214* ^a^
AML with inv(3)(q21.3q26.2) or t(3;3)(q21.3;q26.2)/*GATA2*; *MECOM*(*EVI1*) ^a^
AML with other *MECOM* rearrangements^a^
AML with other rare recurring translocations^a^
AML with t(9;22)(q34.1;q11.2)/*BCR::ABL1* ^b^
AML with mutated *NPM1* ^a^
AML with in‐frame bZIP *CEBPA* mutations^a^
AML with mutated TP53^b^
AML with myelodysplasia‐related cytogenetic abnormalities^b^

^a^ At least 10% of blasts.

^b^ At least 20% of blasts.

Now, since both systems agree that a minimum of 20% blasts is required for the diagnosis of AML with *BCR*::*ABL1* to avoid overlap with chronic myeloid leukemia, this entity will not be addressed further in the following discussion. Furthermore, ICC‐defined AML with *TP53* will also not be discussed because, although the WHO strangely has not included this entity in the list of AML subcategory with defining genetic abnormalities, it recognizes a biallelic inactivation of *TP53* (MDS‐bi*TP53*) as a subtype of MDS and, more importantly, (a) the WHO accepts that this entity should be considered equivalent to AML for therapeutic considerations (and, here, we believe the WHO should simply call these cases AML with *TP53*) and (b) the WHO and the ICC accept that when the blast count is less than 20%, the disease is classified as MDS [3].

For the other entities within the AML subcategory defined by specific genetic abnormalities, it is necessary to apply the criterion that distinguishes between essential and accidental properties:

A blast threshold (20%, 10%, or “percentage increase”) is an essential property of AML subcategory with defining genetic abnormalities just in case it is necessary that AML subcategory with defining genetic abnormalities has a blast threshold (20%, 10%, or “percentage increase”), whereas a blast threshold (20%, 10%, or “percentage increase”) is an accidental property of AML subcategory with defining genetic abnormalities just in case AML subcategory with defining genetic abnormalities has a blast threshold (20%, 10%, or “percentage increase”), but it is possible that AML subcategory with defining genetic abnormalities lacks a blast threshold (20%, 10%, or “percentage increase”).

Is a blast threshold—whether 20%, 10%, or an unspecified “percentage increase”—an essential criterion for diagnosing AML subcategories with defining genetic abnormalities? If so, what is the most appropriate threshold? The following section explores whether these questions can be definitively answered.

## 5 Defining AML Subcategory With Defining Genetic Abnormalities

Due to space constraints, our discussion is limited to those entities for which the evidence appears to be most substantial.

Let us begin with APL with *PML*::*RARA* fusion. The ICC requires a minimum of 10% blasts for the diagnosis. The WHO does not specify a blast cutoff. What is the best definition criterion for this subtype? The point is that, first, even though cases with *PML*::*RARA* rearrangements and less than 10% blasts are rare, they are eventually found in clinical practice [8, 22]. Second, subjective microscopic evaluation influences the enumeration of morphological blasts, and in some cases, it may be challenging to be sure that we are indeed counting blasts (or “blasts equivalents”) [3, 23]. Third, what diagnosis should be made in a patient with, for example, 9% blasts on bone marrow and *PML*::*RARA* rearrangement other than APL with *PML*::*RARA* fusion? The ICC members comment that “many of these cases likely represent *early* AML and could be treated as such if clinically indicated” (our emphasis) [8]. However, rather than providing clarification, this assumption further muddies the waters by introducing a new, undefined concept: “early AML.” What exactly constitutes “early AML”? What is the difference between “early APL” with *PML*::*RARA* and APL with *PML*::*RARA*? Practically speaking, there appears to be none, as both so‐called “early APL” and established APL with *PML::RARA* require prompt treatment. So, things being like this, why apply a 10% blast threshold to define this subtype? In our view, there is no need for a minimum blast percentage for the diagnosis of APL with *PML*::*RARA*. Hence, the WHO definition, which abrogated the blast cutoff in this subtype, offers the most appropriate diagnostic criteria.

With respect to AML with *RUNX1::RUNXT1* and *CBFB::MYH11*, the ICC also considers that “many of these cases likely represent early AML” [8]. But, again, what is “early AML”? Yet, unlike in APL, we are not convinced that the WHO's approach—relying on an “increase of peripheral blood and/or bone marrow blasts”—is the most appropriate for defining these two subtypes, in comparison to ICC's proposal of a minimum blast number of 10%. There are a few reasons why we are uncertain. First, unlike APL, these entities do not constitute true medical emergencies, suggesting that reassessment of the blast percentage could reasonably be deferred. Second, there is no specific treatment with a demonstrated high impact on survival for these AMLs, as there is for APL, although it could be argued that gemtuzumab‐ozogamicin appears to benefit patients with AML with *CBFB::MYH11* [24]. Lastly, and particularly in relation to AML with *RUNX1::RUNXT1*, although some reports suggest that cases with fewer than 10% blasts (and thus previously classified as MDS) may progress rapidly to AML (defined in earlier classifications as exceeding 30% blasts), the number of patients studied remains very limited [25, 26, 27, 28]. It is interesting to speculate that the low percentage of blasts in these patients could be partially explained by the capacity of the blasts to terminal differentiation until neutrophils [27, 28]. In any event, we tend to follow the WHO and assume that individuals with these specific mutations and less than 10% blasts should be diagnosed with AML and, more importantly, treated as AML [22, 25]. However, in the absence of more definitive data, we prefer to assume that currently there are no robust data to favor one or the other classification system.

There are already convincing data showing that there is no need to stipulate a blast threshold for AML cases associated with mutations in the carboxy‐terminal insertion mutations in the nucleophosmin‐1 (*NPM1*), the most common somatic mutations identified in de novo AML. In effect, Patel et al. [29] showed that patients with *NPM1* myeloid neoplasm (less than 20% blasts)—majority represented by cases of MDS with excess blasts, chronic myelomonocytic leukemia (CMML), and MDS/MDS/myeloproliferative (MDS/MPN) neoplasms—had paradoxically inferior overall survival when compared with cases of *NPM1* AML (more than 20% of blasts). (It is important to highlight that, in their series, some cases of *NPM1* myeloid neoplasm had less than 5% of blasts, and some inside this group had blast percentages as low as 1%). A possible explanation for this finding is probably related to the fact that most patients with *NPM1* myeloid neoplasm (33 out of 45 patients) were treated upfront with hypomethylating agents, and 13 of them progressed to AML at a median of 5.9 months. In contrast, none of the three *NPM1* myeloid neoplasm patients who received upfront induction chemotherapy developed AML. These results suggest that upfront hypomethylating agents may have been inadequate for the majority of *NPM1* myeloid neoplasms, and that these cases would likely benefit from more intensive chemotherapy regimens. These findings are consistent with those of Montalban‐Bravo et al. [30] who showed that *NPM1* MDS responds better to chemotherapy than to hypomethylating agents. In summary, there is compelling evidence to support the WHO definition of *NPM1*‐mutated AML, regardless of blast percentage.

The issue with AML harboring *CEBPA* mutations is more complex. The WHO and ICC disagree even with respect to the subcategory which should be incorporated. The WHO includes the entity AML with *CEBPA* mutation: biallelic (bi*CEBPA*) as well as single mutations located in the basic leucine zipper (bZIP) region of the gene (smbZIP::*CEBPA*) [3]. But the ICC included only AML with in‐frame bZIP *CEBPA* mutations [4, 8]. For the WHO, it is necessary at least 20% blasts for the diagnosis because “There is insufficient data to support any change in the blast cutoff criterion for AML with *CEBPA* mutation.” Conversely, the ICC assumes that 10% or more blasts are sufficient for the diagnosis. The two studies alluded by the WHO to give support to the 20% blasts are those of Quintana‐Bustamante et al. [31] and Wen et al. [32]. The first shows that in xenotransplantation model, *CEBPA* mutations (alone or in combination) are insufficient to convert normal human hematopoietic stem cells into leukemic initiating cells. This seems to be confirmed by more recent data [33]. The second study reports *CEBPA* methylation and mutation and their clinical relevance in MDS. The data from Wen et al. [32] is particularly interesting because it shows that neither *CEBPA* methylation nor mutation is associated with survival in MDS patients. However, *CEBPA* mutation was detected in only 4 out of 105 cases, supporting the notion that *CEBPA* mutation occurs rarely in MDS. Zhao et al. [34] recently compared the WHO and ICC criteria in 81 patients with AML with *CEBPA* mutations. The authors concluded that the ICC identifies a more homogeneous group of *CEBPA*‐mutated AML patients with favorable outcomes. This data, however, has limitations related to the blast threshold issue, as the comparison was made in patients with 20% or more blasts. Yet, Huber et al. [21] recently published data from 1451 patients, showing that *CEBPA*‐mutated AML had a median overall survival of 4.1 years based on the WHO criteria, while survival was not reached according to the ICC. Thus, although the current data is insufficient to conclusively resolve the issue of the blast threshold in the presence of *CEBPA* mutation, it seems that, until further evidence emerges, the ICC classification should be admitted [30].

Conversely, we agree with the WHO that for AML involving *KMT2A* and *MECOM*, a blast count under 20% is acceptable, based on studies showing that patients with fewer than 20% blasts and any of these rearrangements exhibit clinical features similar to those with higher blast count. Siddiqui et al. [35] compared cases of *KMT2A* AML with *KMT2A* in MDS and CMML. The authors found that the prognosis of patients with *KMT2A* MDS/CMML was extremely poor, despite lower blast and WBC counts at presentation compared with *KMT2A* AML. Interestingly, the median percentage of blasts in the *KMT2A* MDS/CMML group was 6% (range, 0%–18%), which preliminarily suggests that even cases with fewer than 10% blasts may have a worse prognosis. Given the lack of more significant data at this time, these findings, in conjunction with previous studies showing the poor outcome in MDS with *KMT2A* cases, [36] seem to advocate in favor of the WHO criterion.

Finally, Summerer et al. [37] studied 120 patients with *MECOM* rearrangement: 38 (32%) presented with fewer than 20% bone marrow blasts and were classified as MDS, while 82 (68%) had more than 20% bone marrow blasts and were classified as AML. Kaplan–Meier analysis of MDS patients treated with intensive versus non‐intensive therapy, as well as AML patients treated with intensive versus non‐intensive therapy, revealed similar outcomes regardless of diagnosis, with a more favorable outcome for those receiving intensive treatment. They concluded that the conventional classification of patients with myeloid neoplasms carrying *MECOM* rearrangements into MDS or AML, based on a threshold of 20% bone marrow blasts, does not accurately reflect genetic profiles or clinical outcomes.

## 6 Conclusion

Accurate classifications require accurate definitions. However, while in some domains of science and philosophy the search for definitions is primarily a theoretical endeavor, in medicine, definitions are essential for constructing a reliable nosology.

Which of the two current systems (the WHO or the ICC) best defines AML subcategory with defining genetic abnormalities? The “best” definition is one that enables accurate diagnosis of a serious illness, as misdiagnosis in this context is unacceptable. This implies that (a) the ideal classification system should not categorize an aggressive neoplasm as a “low‐grade” neoplasm (for instance, diagnosing MDS when AML is the more accurate diagnosis, thereby avoiding undertreatment); and (b) it should not classify a less aggressive neoplasm as a more aggressive one (such as giving the diagnosis of AML when the most accurate diagnosis should be MDS, thereby avoiding overtreatment).

Overall, there is substantial agreement between the WHO and ICC classifications, with concordance rates ranging from approximately 83% to 86% [9, 20]. However, their discrepancies remain problematic because, as a recent article appropriately asked: “Is it appropriate to treat a patient with 12% myeloblasts in the marrow and a *CEBPA* mutation with daunorubicin and cytarabine‐based AML induction chemotherapy?” [7]. According to the WHO, the answer is “no,” as this patient would be diagnosed with MDS with increased blasts‐2. In contrast, according to the ICC, the answer is “yes,” since the patient would be classified as having AML with in‐frame bZIP *CEBPA* mutations. In our view, these two systems urgently need to be harmonized.

We believe the first step toward achieving this goal is to establish unified diagnostic criteria for specific entities where the available evidence is compelling. Both the WHO and ICC classifications have strengths and limitations. The WHO defines AML more appropriately than ICC for the entities AML with *PML*::*RARA* rearrangement, with *NPM1* mutation, with *KMT2A* rearrangement, and with *MECOM* rearrangement. Conversely, the ICC provides a more accurate definition for the subtype AML with in‐frame bZIP *CEBPA* mutations. For future classifications, it would be reasonable to define these entities as proposed in Table 3.

**TABLE 3:** Preliminary proposal for a unified WHO and ICC classification.

AML with defining genetic abnormalities	Blast cutoff
APL with *PML::RARA* fusion	Increase peripheral blood and/or bone marrow blasts
AML with *NPM1* mutation	Increase peripheral blood and/or bone marrow blasts
AML with *KMT2A* rearrangement	Increase peripheral blood and/or bone marrow blasts
AML with *MECOM* rearrangement	Increase peripheral blood and/or bone marrow blasts
AML with in‐frame bZIP *CEBPA* mutations	At least 10% of blasts

Unlike the complexity involved in, for instance, finding a definition for CLL—where, in the absence of a pathognomonic genetic lesion, it is necessary to resort to a prototypical theory of concepts to arrive at a satisfactory definition for the disease—[13] the challenge in the case of the AML subcategory with defining genetic abnormalities involves “only” the determination of a blast threshold. This occurs because the concept of AML subcategory with defining genetic abnormalities already incorporates the notion of “principle of the disease mechanism” [15]. The next step, therefore, is to address the following question: Which blast threshold (20%, 10%, or a “percentage increase”) constitutes an essential property for the diagnosis of AML subcategory with defining genetic abnormalities? However, to answer this question, two preliminary questions must first be considered: (1) If the blast threshold alters the disease category, does this have any practical implications for the patient in terms of clinical presentation and prognosis? (2) If the blast threshold alters the disease category, does it affect the patient's access to appropriate treatment?

Medical concepts become obsolete when they no longer serve a practical function; thus, they should not be regarded as rigid entities [16]. Over time, the concept of AML based solely on morphological features and blast count has diminished in utility. Today, this subgroup functions primarily as a diagnosis of exclusion, applied when no defining genetic abnormalities are detected. Notwithstanding, even considering that there is some subjectivity involved in blast enumeration [3, 6], the current question is whether two groups of patients—Group A, with less than 10% blasts, and Group B, with a blast percentage traditionally considered normal for bone marrow (i.e., less than 5%)—but both harboring the same genetic abnormality *X*, exhibit similar therapeutic responses and clinical outcomes.

Future research and classification frameworks should be developed within these perspectives.

## Conflicts of Interest

The authors declare no conflicts of interest.

## Data Availability

The authors have nothing to report.

## Note

i For more on the notion of essential and accidental properties, see Aristotle's works *Topics* 102b5‐26; *Posterior Analytics* 73a34‐16; and *Metaphysics* 1029b13‐15, 1030a1‐6, 1031a12‐25.
